# High Resolution Crystal Structure of the Grb2 SH2 Domain with a Phosphopeptide Derived from CD28

**DOI:** 10.1371/journal.pone.0074482

**Published:** 2013-09-30

**Authors:** Kunitake Higo, Teikichi Ikura, Masayuki Oda, Hisayuki Morii, Jun Takahashi, Ryo Abe, Nobutoshi Ito

**Affiliations:** 1 Research Institute for Biomedical Sciences, Tokyo University of Science, Noda-shi, Chiba, Japan; 2 Medical Research Institute, Tokyo Medical and Dental University, Bunkyo-ku, Tokyo, Japan; 3 Graduate School of Life and Environmental Sciences, Kyoto Prefectural University, Sakyo-ku, Kyoto-shi, Kyoto, Japan; 4 Biomedical Research Institute, National Institute of Advanced Industrial Science and Technology, Tsukuba-shi, Ibaraki, Japan; University of Iowa, United States of America

## Abstract

Src homology 2 (SH2) domains play a critical role in cellular signal transduction. They bind to peptides containing phosphotyrosine (pY) with various specificities that depend on the flanking amino-acid residues. The SH2 domain of growth-factor receptor-bound protein 2 (Grb2) specifically recognizes pY-X-N-X, whereas the SH2 domains in phosphatidylinositol 3-kinase (PI3K) recognize pY-X-X-M. Binding of the pY site in CD28 (pY-M-N-M) by PI3K and Grb2 through their SH2 domains is a key step that triggers the CD28 signal transduction for T cell activation and differentiation. In this study, we determined the crystal structure of the Grb2 SH2 domain in complex with a pY-containing peptide derived from CD28 at 1.35 Å resolution. The peptide was found to adopt a twisted U-type conformation, similar to, but distinct from type-I β-turn. In all previously reported crystal structures, the peptide bound to the Grb2 SH2 domains adopts a type-I β-turn conformation, except those with a proline residue at the pY+3 position. Molecular modeling also suggests that the same peptide bound to PI3K might adopt a very different conformation.

## Introduction

Src homology 2 (SH2) domains are critical components of intracellular proteins that promote signal transduction. SH2 domains recognize phosphotyrosine (pY)-containing sequences in proteins. Growth-factor receptor-bound protein 2 (Grb2) is an adaptor protein that has an SH3-SH2-SH3 domain architecture [1]. The Grb2 SH2 domain mediates activation of the Ras pathway through binding to phosphotyrosyl motifs on either growth factor receptors such as epidermal growth factor receptor or other adaptor proteins such as Shc [2]. Grb2 SH2 specifically binds to the pY-X-N-X consensus sequence where X is any amino acid; however, it binds to pY-(L/V)-N-(V/P) with higher affinity [3], [4]. The selective inhibition of Grb2 SH2 binding to phosphorylated proteins is expected to be useful for the prevention of hyperproliferative diseases.

Three-dimensional structures of Grb2 SH2 in complex with peptides containing pY determined at atomic resolution can be useful for inhibitor development, and several such structures have been reported [4]–[7]. These studies showed that peptides bound to Grb2 SH2 typically adopt a type-I β-turn conformation.

Ligand binding to the CD28 receptor on the T cell surface is a costimulatory signal that acts, along with recognition of the antigen-major histocompatibility complex by the T cell receptor, to trigger full T cell activation and differentiation into effector T cells [8]. A number of signaling molecules such as Grb2 and phosphatidylinositol 3-kinase (PI3K) bind to the cytoplasmic region of CD28 and activate CD28-mediated costimulatory signaling [9], [10]. These molecules bind to CD28 via their SH2 domains primarily to the sequence pY-M-N-M. The consensus Grb2 SH2-binding sequence is pY-X-N-X, whereas the PI3K SH2-binding consensus sequence is pY-X-X-M. CD28 contains the sequence pY-M-N-M in its cytoplasmic region, which enables it to bind both Grb2 SH2 and PI3K SH2 [9]. However, little is known about the molecular details of these interactions.

In this study, we report the crystal structure of Grb2 SH2 in complex with a CD28-derived peptide consisting of 8 amino acids, including the pY-M-N-M sequence, at a resolution of 1.35 Å. This is the first report of the structure of CD28 bound to Grb2 SH2. The high-resolution structure revealed that the bound peptide adopts a conformation similar to, but distinct from the canonical type-I β-turn. Such deviations might exist in other Grb2 SH2/peptide complexes. The possibility that this same peptide adopts a very different conformation when bound to PI3K SH2 is also discussed.

## Materials and Methods

### Expression and purification of the Grb2 SH2 domain

The SH2 domain of human Grb2 (residues 60–152) was expressed in *Escherichia coli* Bl21(DE3) cells as a glutathione *S*-transferase (GST)-fusion protein using the pGEX-4T-1 vector (GE Healthcare) in LB medium containing 100 μg/mL ampicillin. Protein expression was induced with 0.1 mM isopropyl β-D-1-thiogalactopyranoside (IPTG) at 20°C, and the culture was grown for 12 hours. For Grb2 SH2 protein purification, the *E. coli* cell pellet was suspended in lysis buffer (50 mM Tris HCl [pH 8.0] and 150 mM NaCl) and sonicated on ice. After centrifugation, the supernatant was applied to glutathione sepharose 4B beads (GE Healthcare) and eluted with elution buffer (20 mM Tris HCl [pH 8.0], 200 mM NaCl, 500 μM dithiothreitol (DTT), and 10 mM reduced glutathione). The GST protein was separated from Grb2 SH2 by proteolytic cleavage with thrombin (at room temperature, overnight). The Grb2 SH2 protein was further purified by anion-exchange chromatography with a NaCl gradient (0–1.0 M NaCl in 20 mM Tris HCl [pH 8.0]) and gel-filtration chromatography at 4°C. Finally, the purified Grb2 SH2 protein was concentrated to 5 mg/mL in 20 mM Tris HCl (pH 8.0) and 100 mM NaCl.

### Synthesis of the CD28-derived peptide

The 8-residue phosphopeptide, S-D-pY-M-N-M-T-P, which corresponds to residues 189–196 of human CD28, was synthesized by the Fmoc solid-phase method with a PSSM8 peptide synthesizer (Shimadzu Corp.). The C-terminus is a carboxyamide group prepared with Fmoc-NH-SAL-PEG resin (Watanabe Chemicals). Phosphorylated tyrosine was incorporated at the specific position by using *O*-monobenzyl-protected Fmoc-phosphotyrosine (Fmoc-Tyr(PO(OBzl)OH)-OH) [11]. After completion of chain-elongation, the products were cleaved using a mixture of trifluoroacetic acid, 1,2-ethanedithiol, tri-isopropyl silane, and water (86∶6∶6∶2). The peptides were precipitated with diethyl ether, purified by reverse-phase HPLC using YMC-Pack-Pro-C18 column (YMC Co., Ltd.), and verified by mass spectrometry (Shimadzu QP-8000α). Phosphorylation was confirmed by an 8-nm blue shift of the absorption band for tyrosine.

### Crystallization of the Grb2 SH2/CD28 peptide complex

The crystals of the Grb2 SH2/CD28 peptide complex were obtained by the hanging-drop method. Initial screening was performed with Crystal Screen and Crystal Screen 2 (Hampton Research Inc.), which produced small crystals. After refining the conditions, rod-like crystals, up to 200 μm long, were obtained in 100 mM HEPES (pH 7.5), 1.25 M sodium acetate, and 100 mM cadmium sulfate.

### Data collection, structure determination, and refinement

Diffraction data was collected from a single crystal at Beamline NW12A of the Photon Factory (Tsukuba, Japan) at 100K. The diffraction data were integrated and scaled using HKL2000 (HKL Research Inc.). The space group was *P*6_1_22 (*a* = 59.0 Å, *b* = 59.0 Å, *c* = 117.1 Å) and the asymmetric unit contained a single Grb2 SH2/CD28 peptide complex.

Structure determination and refinement was performed using the CCP4 suite [12]. The structure was solved with PHASER [13] by molecular replacement using another previously reported Grb2 SH2 structure [14]. The structure was refined using REFMAC [15] with restrained anisotropic temperature factors. The graphics program Coot was used for model building [16]. In the last cycle of the refinement, the positional restraints for the phosphotyrosine side-chain were removed to allow the diffraction data to determine its structure. The figures were prepared using Discovery Studio (Accelrys Inc.) and Molscript [17].

Some statistics for data collection and structure refinement are shown in Table 1. The coordinates and structural data for the complex have been deposited in the Protein Data Bank (PDB ID: 3WA4).

**Table 1:** Statistics for data collection and structure refinement.

Data collection		
Spacegroup		*P*6_1_22
Unit cell parameters		
* a, b, c* (Å)		59.0, 59.0, 117.1
Resolution (Å)		50–1.35 (1.37–1.35) *
* R*_sym_		0.057 (0.498)
Completeness (%)		97.7 (98.4)
Redundancy		20.6 (21.2)
Refinement		
Resolution (Å)	50–1.35	
Number of reflections	26430	
R_work_/R_free_	0.176/0.209	
Number of non-hydrogen atoms	938	
(Protein atoms)	869	
(Ion atoms)	5	
(Water atoms)	64	
RMS deviations from ideal values		
Bond length (Å)	0.020	
Bond angles (degree)	2.106	
Average B-factor of protein atoms (Å^2^)	19.0	

* Values shown in parentheses are for the highest-resolution shell.

## Results

**Figure 1:**
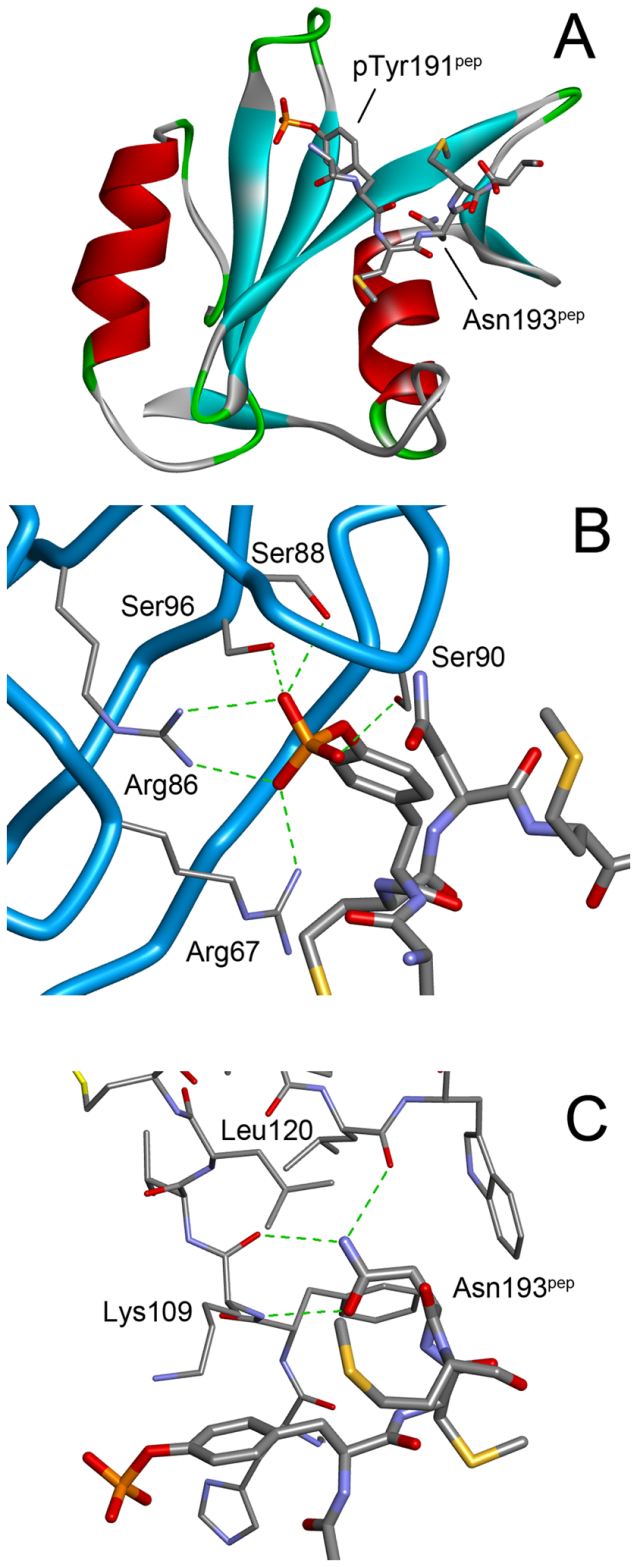
The structure of the Grb2 SH2 domain in complex with a CD28-derived peptide. (A) The overall structure. Grb2 SH2 is shown as a cartoon model, whereas the peptide is shown as a stick model. (B) The interactions between the phosphotyrosine, pTyr191^pep^, and the SH2 domain. The main-chain trace of the SH2 domain is shown as blue tubes with the side-chains of some key residues in thin sticks. The phosphopeptides are shown as thick stick models. The green dashed lines indicate hydrogen bonds. (C) The interactions between the conserved asparagine, Asn193^pep^, of the peptide (thick sticks) and the SH2 domain (thin sticks).

In general, the folds of the Grb2 SH2 domain were essentially the same as those previously reported; consisting of a central, antiparallel β-sheet flanked by 2 α-helices (Fig. 1A) [4]–[7]. The conformation of Trp121 of Grb2 SH2 was the same as other peptide-bound structures with a χ_1_ rotation of approximately 120° compared to the peptide-free structure [7]. The phosphorylated CD28 peptide binds to the Grb2 SH2 recognition site across the exposed edge of the central β-sheet. The phosphotyrosine is located between the β-sheet and the amino-terminal α-helix, and is recognized by a number of residues (Fig. 1B&C). The phosphate moiety of the phosphotyrosine (pTyr191^pep^, the amino acid residues of the CD28-derived peptide are denoted with a “pep” suffix hereafter) directly interacts with the side chains of Arg67, Arg86, Ser88, Ser90, and Ser96. The side-chain of Asn193^pep^ at the pY+2 position forms a pair of hydrogen bonds with the main-chain amide and carbonyl groups of Lys109. Another hydrogen bond is observed between N_δ2_ of Asn193^pep^ and the main-chain O of Leu120. These interactions involving the conserved pTyr and Asn have also been observed in other Grb2/peptide complexes [4]–[7].

The 2 methionine residues, which are unique to the CD28-derived peptide, appear to contribute to the binding mainly through hydrophobic interactions. The side chain of Met192^pep^, at the pY+1 position, is close to the benzene ring of Phe108 and the alkyl of Gln106. The side chain of Met194^pep^, at the pY+3 position, interacts with Leu111 and Lys109 as well as the phosphotyrosine, although these interactions seem weaker than those of Met192^pep^ as suggested by their higher temperature factors (the average temperature factors of the side chain atoms are 27.0 Å^2^ and 38.6 Å^2^ for Met192^pep^ and Met194^pep^, respectively).

**Figure 2:**
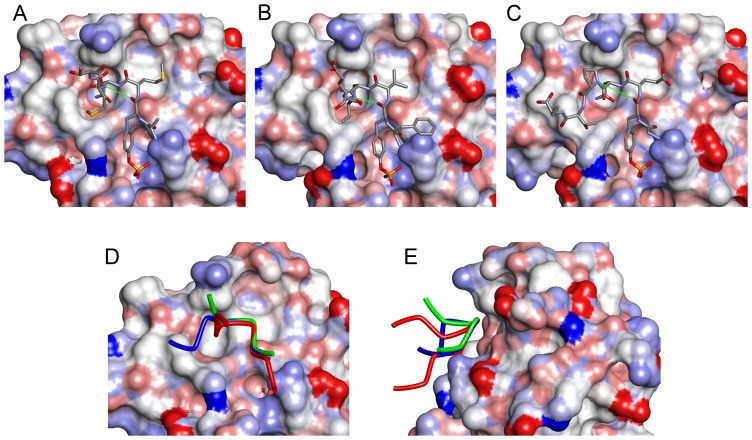
Comparison of the structures of phosphopeptides bound to Grb2 SH2. (A) CD28 (present work, D-pY-M-N-M-T). (B) BCR-Abl (a typical type-I β-turn, PDB ID: 1BMB, F-pY-V-N-V-E) (C) AICD (with a Pro residue at the pY+3 position, PDB ID: 3MXC, G-pY-E-N-P-T-Y). The SH2 domains are shown as surface models, whereas the phosphopeptides are shown as stick models. The thin green lines indicate the distance between the main-chain O of pY and the main-chain N of pY+3, which form a hydrogen bond in the type-I β-turn. The side-chains of some flanking residues are missing due to their weak electron density. (D) Superposition of the 3 peptides. The tubes represent the main-chain traces of CD28 (green), BCR-Abl (red), and AICD (blue). (E) Superposition of CD28, BCR-Abl, and AICD as in (D) but vertically rotated by approximately 90°.

The peptide adopts a bent conformation similar to the type-I β-turn, which is the canonical conformation of peptides bound to Grb2 SH2 [5], [18], [19]. However, the hallmark hydrogen bond between the main-chain oxygen of pY and the main-chain nitrogen of the pY+3 residue (Met194^pep^ in our structure) is not formed (Fig. 2A). Not only are they separated by greater than 3.7 Å, but the direction of the N-H bond, assuming an ordinary structure for the amide group, does not point toward the carbonyl oxygen, making the presence of the hydrogen bond unlikely. Comparison of the main-chain torsion angles φ and ψ shows that the difference between this structure and the type-I β-turn is mainly caused by the ψ angle of the conserved Asn residue at pY+2 (Table 2). In type-I β-turns, this angle should be close to 0°; however, in our structure, it is approximately 40°. Consequently, the CD28-derived peptide is slightly lifted away from Grb2 SH2, making it a more “twisted” conformation than a canonical type-I β-turn (Fig. 2D). This twist also creates more space between the peptide and the protein, and accommodates the side chain of Met194^pep^.

**Table 2:** Main-chain torsion angles (φ/ψ) of the phosphopeptide bound to the Grb2 SH2 domain and their amino acid sequences^a^.

PDB ID	Resolution (Å)	pY+1	pY+2 [N]	pY+3	O–N distance^b^ (Å)
1BMB	1.8	–52.9/–33.4 [V]	–99.6/14.2	[V]	3.03
1BM2	2.1	–58.9/–44.5 [V]	–83.0/–12.6	[V]	3.22
1JYR	1.55	–59.0/–32.3 [V]	–103.8/14.9	[V]	3.11
1TZE	2.1	–54.9/–28.9 [V]	–103.2/12.8	[V]	3.02
1ZFP	1.8	–61.8/–43.4 [I]	–88.0/19.8	[Q]	3.37
3N8M	2.0	–52.4/–35.0 [V]	–100.7/10.9	[V]	3.07
CD28 c	1.35	–72.7/–21.5 [M]	–103.6/39.8	[M]	3.71
β-turn^d^	–	–60/–30	–90/0	–	−
3MXC	2.0	–67.3/–32.9 [E]	–99.2/127.0	[P]	5.10
3MXY	2.3	–60.6/–40.3 [V]	–92.8/142.8	[P]	5.11

a The angles are given in degree. The residues in the pY+1 and pY+3 positions are shown as one-letter codes in square brackets.

b The distance between the main-chain O of pY and the main-chain N of pY+3, which form a hydrogen bond in type-I β-turn.

c The structure reported in this work.

d Theoretically idealized values for type-I β-turn.

**Figure 3:**
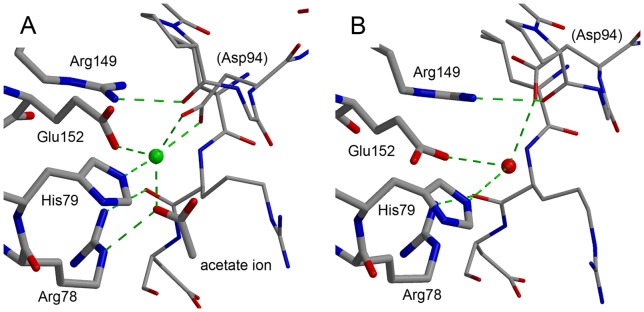
The cadmium binding site. (A) The cadmium binding site located between 2 neighboring molecules in the crystal. The cadmium ion is shown as a green sphere. The Grb2 SH2 molecule is shown in thick lines, whereas a symmetrically-related molecule is shown in thin lines. Green dashed lines indicated the coordinate and hydrogen bonds. (B) The corresponding site in Grb2 SH2/AICD (PDB ID: 3MXC). The red sphere represents a water molecule.

A very strong electron density, which was interpreted as a cadmium ion, was observed between 2 molecules in the crystal lattice. This ion is coordinated by the Nε_2_ of His79 and a carboxyl oxygen of Glu152 of one Grb2 molecule and 2 carboxyl oxygens of Asp94 in a neighboring molecule. An acetate ion, which was required for the crystallization, also coordinated to it (Fig. 3A).

In our crystallization trials, the addition of cadmium sulfate markedly improved the appearance and diffraction quality of the crystals. Among the previously reported Grb2 SH2 crystal structures, 2 (PDB ID: 3MXC and 3MXY) have the same space groups and similar unit cell parameters as our structure [20]. These 2 structures are Grb2 SH2/amyloid precursor intracellular C-terminal domain (AICD)-derived peptide structures. A comparison of these Grb2 SH2/AICD structures with ours revealed that their crystal packing is very similar. Yet the resolutions of the Grb2 SH2/AICD 2 structures, 2.0 Å and 2.3 Å, are much lower than that of our structure at 1.35 Å. In these other structures, the cadmium binding site is occupied by a water molecule that forms some hydrogen bonds (Fig. 3B). The presence of the cadmium ion appears to have increased the number of polar interactions between the 2 molecules. It may also have contributed to the improved crystal quality by replacing the intermolecular network of hydrogen bonds with stronger coordinate bonds.

**Figure 4:**
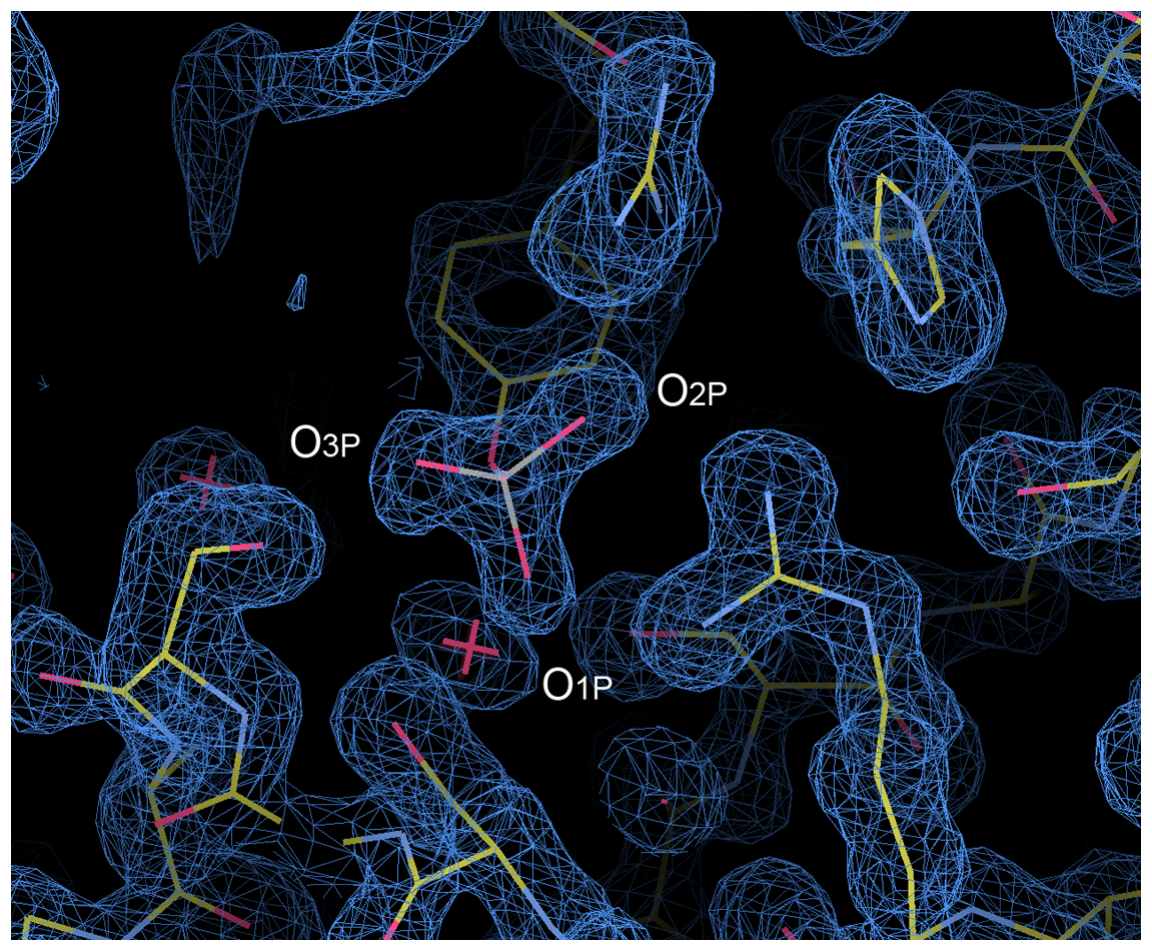
The 2Fo–Fc map around the phosphate group of the phosphotyrosine residue, pTyr191^pep^. The contour level is set to 1.2 σ, where σ is the root-mean-square deviation of the electron density.

The high resolution of the structure presented here allowed us to determine the geometry of the phosphotyrosine in detail (Fig. 4 & Table 3). In the very last cycle of the structure refinement, the positional constraints for the side-chain atoms of the phosphotyrosine were removed to make the most of the experimental data and investigate its geometry. Two of the three bond angles between the phenol oxygen atom and the phosphate oxygen atoms (O_η_–P–O_nP_, where n = 1, 2, or 3) are smaller than 109.5°, the theoretical value for ideal tetrahedral geometry, indicating that the phosphate oxygen atoms are somewhat more “spread up” than typical tetrahedral geometry. In addition, the 3 phosphate oxygens are in an asymmetrical arrangement, deviating from an equilateral triangle. To match one phosphate oxygen atom to another in a symmetrical arrangement, the rotation angle around the phenol oxygen-phosphorus bond (O_η_–P) should be 120°. However, that is not the case for the phosphotyrosine in our structure. The difference between the maximum and minimum rotation angles is greater than 10°. Similar asymmetry is also observed in the phosphotyrosine molecule structures reported by small-molecule X-ray crystallography, whose resolution is 0.77 Å [21].

**Table 3:** Selected bond angles and torsion angles of the phosphate group of the phosphotyrosine (degrees).

	CD28 a	Small molecule crystallography b		REFMAC c
		A	B	
*Bond angles*				
C_ζ_–O_η_–P	129.6	125.5	122.8	120.0
O_η_–P–O_1P_	94.7	103.2	108.3	108.2
O_η_–P–O_2P_	106.4	104.5	106.8	108.2
O_η_–P–O_3P_	110.8	106.9	103.7	108.2
(Average of O_η_–P–O_nP_)	(104.0)	(104.9)	(106.3)	(108.2)
O_1P_–P–O_2P_	112.9	106.5	111.0	119.9
O_2P_–P–O_3P_	115.6	121.5	111.4	119.9
O_3P_–P–O_1P_	114.1	112.5	115.0	119.9
*Torsion angles*				
C_ζ_–O_η_–P–O_1P_	–149.0	–85.4	61.2	–
C_ζ_–O_η_–P–O_2P_	–33.4	25.7	178.8	–
C_ζ_–O_η_–P–O_3P_	93.0	155.6	–58.5	–
(Rotation O_1P_ →O_2P_)	(115.6)	(111.1)	(117.6)	(120.0)
(Rotation O_2P_ →O_3P_)	(126.4)	(129.9)	(122.7)	(120.0)
(Rotation O_3P_ →O_1P_)	(118.0)	(119.0)	(119.7)	(120.0)

a Present work.

b Results from small-molecule X-ray crystallography [21]. As the asymmetric unit contains 2 phosphotyrosine molecules, denoted A and B, the values for both are shown.

c Values from the dictionary files of REFMAC.

## Discussion

Here, we reported the crystal structure of the Grb2 SH2 domain in complex with a phosphorylated peptide derived from CD28. The structure was determined at a resolution of 1.35 Å, the highest among the Grb2 SH2 domain structures reported to date. The structure revealed a unique feature of Grb2-SH2 binding to the CD28-derived peptide. In all previously reported Grb2 SH2/peptide complex structures, the peptide containing the phosphotyrosine residue adopted a type-I β-turn (Fig 2B) [19], except for the AICD-derived peptides in 2 Grb2 SH2/AICD structures [20]. The AICD-derived peptides have a proline residue at the pY+3 position and are incapable of forming a β-turn because proline does not have the amide hydrogen required for the characteristic hydrogen bond (Fig. 2C). The CD28-derived peptide reported here has a methionine residue at the pY+3 position, which is capable of forming the hydrogen bond. However, its structure is not a canonical type-I β-turn. Although it has a U-shaped conformation, similar to the β-turn, it is somewhat twisted and lacks the key hydrogen bond. This is the first such example.

The ψ angle of the asparagine at the pY+2 position seems to be largely responsible for this deviation from the type-I β-turn (Table 2). The larger value of the ψ angle moves the pY+3 residue slightly away from Grb2 SH2, making room for the side-chain of Met193^pep^. In another words, this bulky side-chain lifted the peptide away from Grb2 SH2. The Grb2 SH2/peptide complex structures reported thus far have relatively small residues at the pY+3 position. Loss of the hydrogen bond may be compensated by the hydrophobic interaction between the methionine of the peptide and Grb2 SH2.

The φ angle of Met192^pep^ at the pY+1 position in our structure also deviates from that of the other Grb2 SH-bound peptides with type-I β-turns although the difference is smaller than that of the ψ angle discussed above. These 2 angles are complementary for maintaining the hallmark hydrogen bond, and the change in the φ angle of Met192^pep^ compensates for deviation of the ψ angle of Asn193^pep^ to some extent, keeping the 2 the main-chain oxygens of pTyr191^pep^ relatively close to the main-chain nitrogen of Met194^pep^. The peptide may transiently adopt a type-I β-turn conformation in solution before binding to Grb2 SH2.

It is tempting to speculate that other peptides containing a residue with a large side chain may also adopt the twisted U-shape conformation, rather than the canonical type-I β-turn. Interestingly, when an epidermal growth factor (EGF)-derived peptide, which has a relatively large glutamine residue at the pY+3 position, is bound to Grb2 SH2 (PDB ID: 1ZFP), it adopts a conformation between the type-I β-turn and the twisted U-shape found in our structure [22]; both its ψ angle value (19.8°) and the O–N distance (3.37 Å) are intermediate between those of the type I β-turn and the twisted U-shape (Table 2).

**Figure 5:**
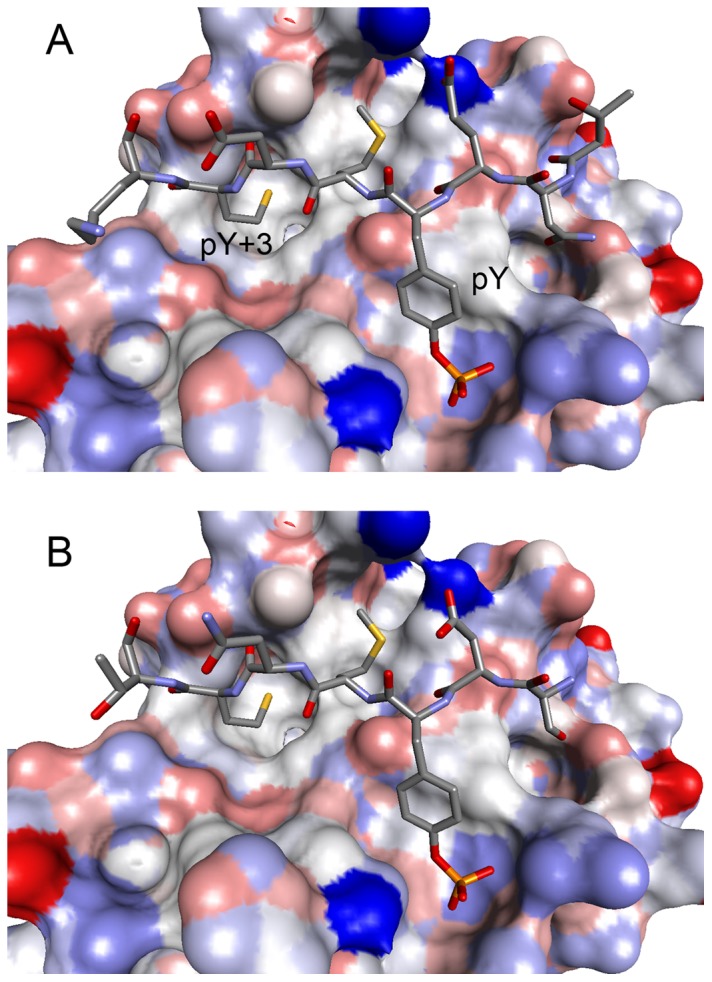
A model structure of the CD28-derived peptide bound to PI3K N. (A) The crystal structure of the amino-terminal SH2 domain of PI3K (PI3K N SH2) with a phosphopeptide derived from c-Kit (T-N-E-pY-M-D-M-K) and (B) a molecular model of PI3K N SH2 with the CD28-derived peptide (S-D-pY-M-N-M-T). The SH2 domains are shown as surface models, whereas the phosphopeptides are shown as stick models.

In this study, the CD28-derived phosphopeptide binds to Grb2 SH2 in the twisted U-shape conformation. This phosphopeptide also binds to the SH2 domains of phosphatidylinositol 3-kinase (PI3K), whose consensus binding motif is pY-X-X-M. The crystal structure of the amino-terminal SH2 domain of PI3K (PI3K N SH2) containing a phosphopeptide derived from c-Kit [23] offers good insight into the interaction between the CD28-derived peptide and PI3K SH2 (Fig. 5A). The sequence of the c-Kit-derived peptide is TNE(pY)MDMKPGV, and this peptide bound to the SH2 domain in an extended conformation. A molecular model of the CD28-derived peptide bound to PI3K N SH2 can be made by simply replacing the sequence of the c-Kit peptide with that of the CD28-derived peptide, SD(pY)MNMT. The model would preserve most of the key protein-peptide interactions from the pY to pY+3 potions, with no obvious unfavorable interactions (Fig 5B). Therefore, one can expect that the CD28-derived peptide changes conformation in a receptor-dependent manner. The β-turn (or twisted U-shape) conformation of the Grb2 SH2-bound peptide positions the pY+2 residue close to the protein, making this residue highly conserved, whereas the extended conformation of the PI3K N SH2-bound peptide exposes the pY+2 residue to solvent, and it has no strong interactions with the protein. Instead, the residues in the pY+1 and pY+3 positions strongly interact with the protein. CD28 exploits the differences between the molecular recognition of pY by the PIK3 and Grb2 SH2 domains to enable binding to both proteins via a single pY site.
